# Proteomic Profiling for Peritoneal Dialysate: Differential Protein Expression in Diabetes Mellitus

**DOI:** 10.1155/2013/642964

**Published:** 2013-05-28

**Authors:** Ming-Hui Yang, Hsien-Yi Wang, Chi-Yu Lu, Wan-Chi Tsai, Po-Chiao Lin, Shih-Bin Su, Yu-Chang Tyan

**Affiliations:** ^1^Department of Chemical and Materials Engineering, National Yunlin University of Science and Technology, Yunlin 640, Taiwan; ^2^Department of Nephrology, Chi-Mei Medical Center, Tainan 710, Taiwan; ^3^Department of Sport Management, College of Leisure and Recreation Management, Chia Nan University of Pharmacy and Science, Tainan 717, Taiwan; ^4^Department of Biochemistry, College of Medicine, Kaohsiung Medical University, Kaohsiung 807, Taiwan; ^5^Department of Medical Laboratory Science and Biotechnology, Kaohsiung Medical University, Kaohsiung 807, Taiwan; ^6^Department of Laboratory Medicine, Kaohsiung Medical University Hospital, Kaohsiung 807, Taiwan; ^7^Department of Chemistry, National Sun Yat-Sen University, Kaohsiung 804, Taiwan; ^8^National Sun Yat-Sen University-Kaohsiung Medical University Joint Research Center, Kaohsiung 807, Taiwan; ^9^Department of Family Medicine, Chi-Mei Medical Center, Tainan 710, Taiwan; ^10^Department of Medical Imaging and Radiological Sciences, Kaohsiung Medical University, 100 Shi-Chuan 1st Road, Kaohsiung 807, Taiwan

## Abstract

Peritoneal dialysis (PD) is an increasingly accepted modality of renal replacement therapy. It provides the advantages of having a flexible lifestyle, stable hemodynamics, and better preservation of residual renal function. To enhance our understanding of the peritoneal dialysate of diabetes mellitus (DM), peritoneal dialysate proteins were identified by two-dimensional gel electrophoresis (2DE) combined with reverse-phase nano-ultra performance liquid chromatography electrospray ionization tandem mass spectrometry (RP-nano-UPLC-ESI-MS/MS) followed by peptide fragmentation patterning. To validate the differential proteins, ELISA and Western blotting analyses were applied to detect candidate proteins that may be related to DM. We performed 2DE on the peritoneal dialysate samples, with detection of more than 300 spots. From this, 13 spots were excised, in-gel digested, and identified by RP-nano-UPLC-ESI-MS/MS. Ten of these showed significant differential expression between the DM and chronic glomerulonephritis (CGN) peritoneal dialysate samples. In this study, we conducted a comparative proteomic study on these two groups of dialysate that may provide evidence for understanding the different peritoneal protein changes. These proteins may not be new biomarkers; however, they may indicate a situation for possible drug treatment and can be the predictors of peritonitis for a validation study in the future.

## 1. Introduction

Peritoneal dialysis (PD) is a treatment for patients with severe chronic kidney disease such as renal failure or renal insufficiency. The process uses the patient's peritoneum in the abdomen or coelom as a membrane across which fluids and dissolved substances (electrolytes, urea, glucose, proteins, and other small molecules) are exchanged between the blood and the peritoneal fluid (to which a high-glucose solution has been infused) [1]. PD is today an increasingly accepted modality of renal replacement therapy. The problem with this therapy is that, upon prolonged treatment, peritoneal fibrosis with subsequent loss of ultrafiltration (UF) ability and fluid overload could occur, which will result in the necessity of discontinuation of PD. An additional problem would be also the onset of peritonitis, which might occur upon prolonged treatment [2]. Recurrent peritonitis and bioincompatibility of the peritoneal dialysate (e.g., high blood glucose, high osmolality, acidic pH, and elevated glucose degradation products) are the leading mechanisms attributed to peritoneal fibrosis [3]. Increasing levels of the dialysate/plasma creatinine ratio (D/P Cr) on the peritoneal equilibrium test (PET) indirectly reflect possible peritoneal fibrosis-related UF failure [4]. So far, invasive peritoneal biopsy is the only method in current daily practice which can provide both definite evidence and prediction of peritoneal fibrosis. Therefore, a noninvasive diagnostic method is urgently needed. Several studies have been focused on the analysis of spent dialysate composition, including biochemistry profiles [5], cytokines [6], or proteomics [7, 8]. Among these, proteins are the final biological factors for performing biochemical reactions and physiological responses. Several studies have reported the unique proteomic profiling of pleural effusion [9, 10] or urine [11, 12]. Analyzing proteomic presentation of the dialysate may provide clues for both understanding the mechanisms of peritoneal damages and noninvasively predicting the changes of peritoneal transport characters. Brauner et al. reported that the increased amount of novel protein (daintain/allograft inflammatory factor-1, AIF-1) is detected during the early stage of PD initiation and peritonitis [13]. Another study also reported that the elevation of vitamin D-binding protein, haptoglobin, and *α*-2 microglobulin was noted in the diabetes mellitus (DM) dialysate [14]. Furthermore, the target proteomic changes may function as novel biomarkers for disease monitoring or detection in vitro.

Finding biomarkers in the complex biological matrix of proteins in the peritoneal dialysate for DM is a challenge. Although there are numerous proteins, lipids, and metabolic products in dialysate samples, disease biomarkers often appear at low concentrations. High-abundance proteins such as albumin, IgG, and fibrinogen are dominant and may repress the signals of the low-abundance proteins. In recent years, several maturing analytical tools have been applied in proteomic research, such as two-dimensional gel electrophoresis (2DE) and mass spectrometry analysis, which have provided much information on body fluid analysis [14, 15]. 

PD is one modality of the renal replacement therapy and is adopted increasingly all over the world due to more stable hemodynamic and biochemistry level and less cost than hemodialysis. End-stage renal disease due to DM is not contraindicated for peritoneal dialysis. DM and chronic glomerulonephritis (CGN) are the most common etiologies of end-stage renal disease (ESRD) receiving maintenance peritoneal dialysis [1]. In this proteomic study, we assembled a list of proteins observed in peritoneal dialysates from DM and CGN patients. To enhance our understanding of the DM proteome, the peritoneal dialysate proteins were identified by 2DE and nano-ultra performance liquid chromatography electrospray ionization tandem mass spectrometry (nano-UPLC-ESI-MS/MS) followed by peptide fragmentation patterning. This study is designed to establish an optimal technique for a proteomic map of DM proteins. The database provides not only analysis of PD proteins from DM patients but also suggests potential diagnostic protein biomarkers for further investigation.

## 2. Materials and Methods

### 2.1. Peritoneal Dialysate Collection

Enrolled patients received regular peritoneal dialysis for more than three months, without recent major comorbidity, surgery, or hospital admission within 1 month before the study. Human peritoneal dialysate specimens were collected after standard PET from 12 DM and 12 CGN patients. Two groups of patients were matched by age and gender. The Human Experiment and Ethics Committee of the Chi-Mei Medical Center approved the study protocol, and all subjects signed informed consents to the study performed. This study was approved by the Institutional Research Board and executed according to the Helsinki Declaration Principles. Written informed consents were received from all participating patients. Each peritoneal dialysate specimen (100 mL) was centrifuged at 1000 ×g at 4°C for 10 min. The supernatant was stored in polypropylene tubes at −80°C. The protein concentrations of the peritoneal dialysate samples were measured using a fluorescence-based protein quantification detection kit (Quant-iT Fluorometer, Qubit Protein Assay Kit, Q33212, Invitrogen).

### 2.2. Two-Dimensional Gel Electrophoresis (2DE)

The protein in the peritoneal dialysates (containing 120 *μ*g protein) was precipitated with 10% w/v trichloroacetic acid (TCA) in acetone. The precipitates were washed with acetone three times and dissolved in an isoelectric focusing (IEF) rehydration buffer for 2DE analysis. IEF strips (pH 3–10, IPGphor, Amersham Biosciences, Uppsala, Sweden) were developed through a stepwise incremental voltage program: 30 V for 16 hr, 500 V for 1 hr, 1000 V for 1 hr, and 8000 V for 4 hr, with a total power of 34 kV hr. Then the strips were subjected to a two-step equilibration in buffers: the first step containing 6 M urea, 30% glycerol, 2% SDS, and 50 mM Tris-HCl (pH 8.8) with 1% w/v dithiothreitol (DTT, Affymetrix/USB, 15395) and for the second step, with 2.5% w/v iodoacetamide (IAA, Amersham Biosciences, RPN6302V). The strips were then transferred onto the second-dimensional SDS-PAGE equipment and developed on 1.0 mm thick gradient (8–16%) polyacrylamide gels at 10°C. 

### 2.3. Silver Staining

The gels were fixed in 40% v/v ethanol and 10% v/v acetic acid in water overnight and then incubated in a buffer solution containing 30% v/v ethanol, 6.8% w/v sodium acetate, and 0.312% w/v sodium thiosulfate for 30 min. After washing three times in water for five minutes each, the gels were stained for 30 minutes in 0.25% w/v silver nitrate solution containing 0.02% w/v formaldehyde. Development was performed for 10 min in a solution consisting of 2.5% w/v sodium carbonate and 0.01% w/v formaldehyde. Acetic acid solution (5% v/v) was used to stop the development; then the stained gels were rinsed three times in water for five minutes each.

### 2.4. Image Acquisition and Analysis

The stained gels were scanned using an ImageScanner and LabScan 3.00 software (Amersham Biosciences). Image analysis was carried out using the ImageMaster 2D, version 2002.1 (Amersham Biosciences). In this study, we performed 2DE on three repeats of each sample, respectively. To prevent variation between different 2DE gels, all volumes of protein spots in the 2DE map were normalized by using the ratio of each spot over the sum.

### 2.5. Statistics and Tryptic In-Gel Digestion

Protein spots were selected by using the criteria: protein expressions are a twofold difference between DM and CGN patients (greater than twofold or less then 50%). Statistical significance was determined by using the two-tailed Fisher exact test at *P* < 0.05. The protein spots were excised manually, reduced, alkylated, and then digested using sequence grade trypsin (V511A, Promega) by standard protocols [16].

### 2.6. Protein Identification

The tryptic digests of the proteins were separated and identified using a flow rate of 400 nL/min with an RP-nano-UPLC system (nanoACQUITY UPLC, Waters, Milford, MA, USA) coupled to an ion trap mass spectrometer (LTQ Orbitrap Discovery Hybrid FTMS, Thermo, San Jose, CA, USA) equipped with an electrospray ionization source. For RP-nano-UPLC-ESI-MS/MS, a sample (2 *μ*L) of the desired peptide digest was loaded into the reverse phase column (Symmetry C18, 5 *μ*m, 180 *μ*m × 20 mm) by an autosampler. The RP separation was performed using a linear acetonitrile gradient from 99% buffer A (100% D.I. water/0.1% formic acid) to 85% buffer B (100% acetonitrile/0.1% formic acid) in 45 min using the micropump. The separation was performed on a C18 microcapillary column (BEH C18, 1.7 *μ*m, 75 *μ*m × 100 mm) using the nanoseparation system. As peptides eluted from the microcapillary column, they were electrosprayed into the ESI-MS/MS with the application of a distal 2.1 kV spraying voltage with heated capillary temperature of 200°C. Each cycle of one full-scan mass spectrum (*m*/*z *400–2000) was followed by four data-dependent tandem mass spectra with collision energy set at 35%.

### 2.7. Database Search

For protein identification, Mascot software (Version 2.2.1, Matrix Science, London, UK) was used to search the Swiss-Prot human protein sequence database. For proteolytic cleavages, only tryptic cleavage was allowed, and the number of maximal internal (missed) cleavage sites was set to two. Variable modifications of cysteine with carboxyamidomethylation, methionine with oxidation, serine/threonine/tyrosine with phosphorylation, and asparagine/glutamine with deamidation were allowed. Mass tolerances of the precursor peptide ion and fragment ion were set to 20 ppm and 1 Da, respectively. Positive protein identifications were defined when the Mowse scores greater than 100 were considered significant (*P* < 0.05). Proteins were initially annotated by similar searches using UniProtKB/Swiss-Prot databases.

### 2.8. Western Blotting

Four proteins, apolipoprotein A-I (APOA1), apolipoprotein A-IV (APOA4), eukaryotic translation initiation factor 4A isoform 1 (EIF4G1), and Zn-alpha2-glycoprotein (AZGP1), were analyzed by Western blotting. Each peritoneal dialysate sample (1 *μ*g/*μ*L, 10 *μ*L) was electrophoresed through a precast gel (NuPAGE Novex 4–12% Bis-Tris Gel, 1.5 mm, 10 wells, Invitrogen, Carlsbad, CA, USA). Proteins were transferred from the gel to a polyvinyl difluoride (PVDF) membrane (Millipore, Bedford, CA, USA) by means of the semidry technique using the Criterion Blotter (Bio-Rad) at 100 V for 60 min and blocked with 5% milk in PBS (adjusted to pH 7.4) containing 0.05% Tween-20. 

The membranes were then incubated overnight with rabbit anti-APOA1 (2840-1, Epitomics), rabbit anti-APOA4 (3109-1, Epitomics), anti-EIF4G1 (E8949-54, Labome), and rabbit anti-AZGP1 (ab117275, Abcam) diluted to 1 *μ*g/mL. After washing, the membrane was incubated with alkaline peroxidase-conjugated affinipure goat anti-rabbit IgG (111-035-003, Immuno Research) diluted 1/10000 for 1 hr. The proteins were detected with an enhanced chemiluminescence (ECL) system. The quantitative analysis of Western blotting was carried out using the ImageQuant-TL-7.0 software, version 2010 (Amersham Biosciences) [17].

### 2.9. ELISA Assay

Each peritoneal dialysate sample was analyzed for the concentrations of APOA1 (E-80AP1, Immunology Consultants Laboratory), alpha-1-microglobulin/bikunin preproprotein (AMBP) (E90217Hu, Uscn Life Science Inc.), mutant retinol-binding protein (RBP4) (E90929Hu, Uscn Life Science Inc.), and haptoglobin (HP) (E-80HPT, Immunology Consultants Laboratory) in duplicate, using commercially available enzyme-linked immunosorbent assay (ELISA) kits. The protein concentrations were tested by standard protocols, according to the suggestion of the manufacturers. The model of ELISA reader was Multiskan EX, which was made by Thermo Scientific (Vantaa, Finland). Statistical significance was determined by using the two-tailed Fisher exact test at *P* < 0.05 and ROC analysis.

## 3. Results and Discussion

Peritoneal dialysis is one of the therapeutic options for ESRD patients. The treatment involves introduction of a high glucose concentration of fluid to the patient peritoneum, which causes waste products in the blood to diffuse across the peritoneal membrane. DM is one of the major diseases treated by PD, in which the high glucose concentration may accelerate damage to the peritoneal membrane by increasing the permeability and decreasing the ultrafiltration efficiency. It will cause peritoneal membrane dysfunction and the patient may have fluid overload-related complications or eventually require hemodialysis. Long-term high blood glucose level will result in multiple organs damage due to either microangiopathy or macroangiopathy. Diabetes is the most common etiology of new uremic patients receiving dialysis therapy. Meanwhile, chronic glomerulonephritis is also one of the leading causes of end-stage renal disease. It presented with long-term proteinuria and/or hematuria and slowly decreasing renal function, predominantly involving kidneys. Comparison of proteomic differentiation in peritoneal dialysis fluid from DM and CGN patients provides opportunity to noninvasively searching evidence or predicting biomarkers of glucotoxicity-related damage of peritoneum. It is impossible to obtain “healthy” control peritoneal dialysate. Thus, we utilized the peritoneal dialysate from CGN patients as control samples and compared them with the DM peritoneal dialysate. In this study, we described the observation of significant differential expression of several proteins in DM peritoneal dialysate.

### 3.1. 2DE Map of Peritoneal Dialysate

**Figure 1:**
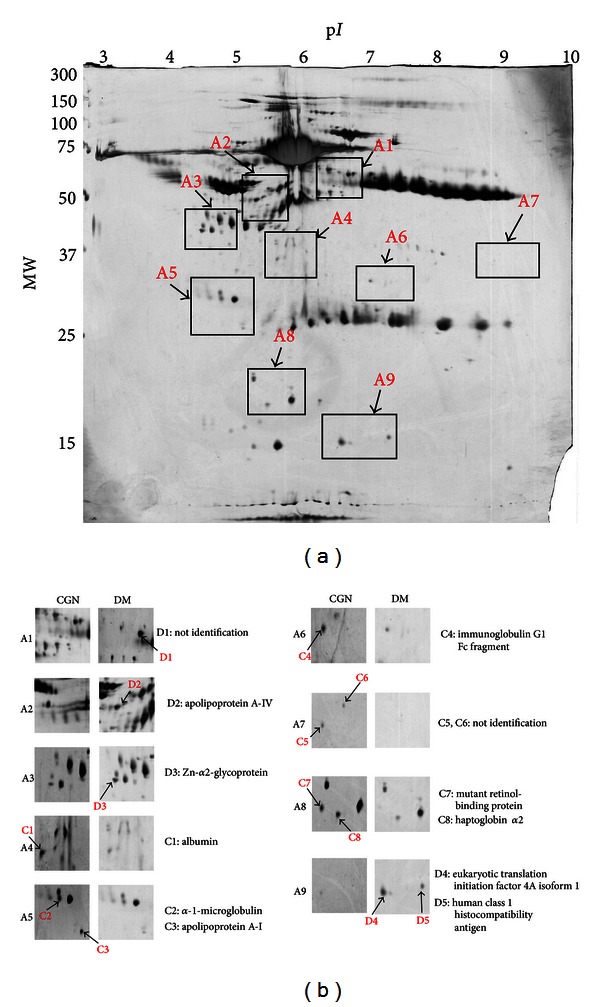
Representative 2DE maps of peritoneal dialysate samples (a) CGN and (b) compared with DM. Peritoneal dialysate samples were separated for 2DE analysis (pH 3–10), and 120 *μ*g protein of each sample was loaded into each gel. The analysis of each sample was repeated in three gels.

We performed 2DE on the peritoneal dialysate from DM and CGN patients, and more than 300 spots were detected using the image analysis software (Figure 1(a)). The protein identifications in the 2DE analysis of the peritoneal dialysate were compared with the 2DE images of normal human plasma (downloadable from the Swiss-Prot/TrEMBL website: http://world-2dpage.expasy.org/swiss-2dpage/docs/publi/inside1995.html). The most abundant proteins in the peritoneal dialysates were matched with the plasma 2DE map (according to the MW, pI, and pattern of spots) such as albumin, microglobulin, haptoglobin, glycoprotein, fibrinogen, and immunoglobulin. 

The criteria of proteins selected were protein expressions of more than twofold difference between the DM and CGN peritoneal dialysate (greater than twofold or less then 50%). Statistical significance was determined by using the two-tailed Fisher exact test at *P* < 0.05. In this study, 13 of the protein spots showed significant differential expression between the DM and CGN peritoneal dialysate samples (Figure 1(b)). However, three of those spots were not identified (D1, C5, and C6 in Figure 1(b)). Ten identified proteins and their functions are listed in Tables 1 and 2. These proteins can be classified into two major functional groups as binding/transport and acute-phase/immune response.

### 3.2. Protein Identification by RP-Nano-UPLC-ESI-MS/MS

The fragmentation spectra from the RP-nano-UPLC-ESI-MS/MS analysis were searched against a nonredundant protein database by MASCOT. When a protein was identified by three or more unique peptides possessing MASCOT scores, no visual assessment of spectra was conducted and the protein was considered present in the sample. All Mascot results were manually confirmed by visual assessment of the MS/MS spectra for overall quality. In addition, the criterion for manual validation reported by Jaffe et al. was used [18]. It requires a readily observable series of at least 4 y ions.

For the ten identified proteins, 4 upregulated and 6 downregulated proteins were found in DM peritoneal dialysate. The ten proteins were positively identified as apolipoprotein A-IV (APOA4), Zn-alpha2-glycoprotein (AZGP1), eukaryotic translation initiation factor 4A isoform 1 (EIF4G1), human class I histocompatibility antigen (HLA-A) (upregulation), albumin (ALB), alpha-1-microglobulin/bikunin preproprotein (AMBP), apolipoprotein A-I (APOA1), immunoglobulin G1 Fc fragment (IGHG1), mutant retinol-binding protein (RBP4), haptoglobin alpha2 (HP) (downregulation), which may serve as potential protein indicators of DM.

### 3.3. DM-Related Proteins

To validate the differential proteins identified by nano-UPLC-ESI-MS/MS, ELISA and Western blotting analyses were applied to detect candidate proteins that may be related to DM. In this study, 6 proteins were selected and confirmed by ELISA or Western blotting. 

**Figure 2:**
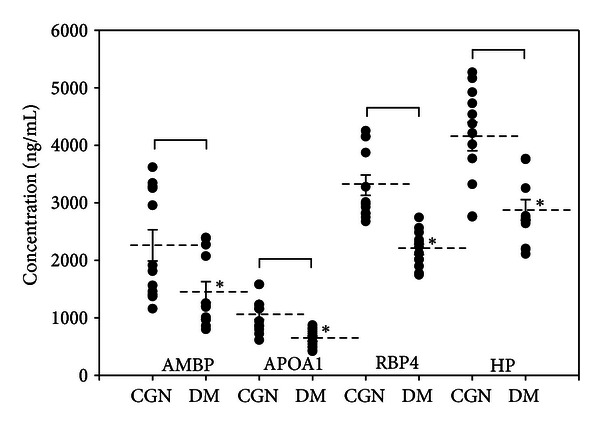
Confirmation of AMBP, APOA1, RBP4, and HP expressions by ELISA analysis (*n* = 12, 3 repeats, *P < 0.05). Scatter plots of the individual values for each protein are shown.

**Figure 3:**
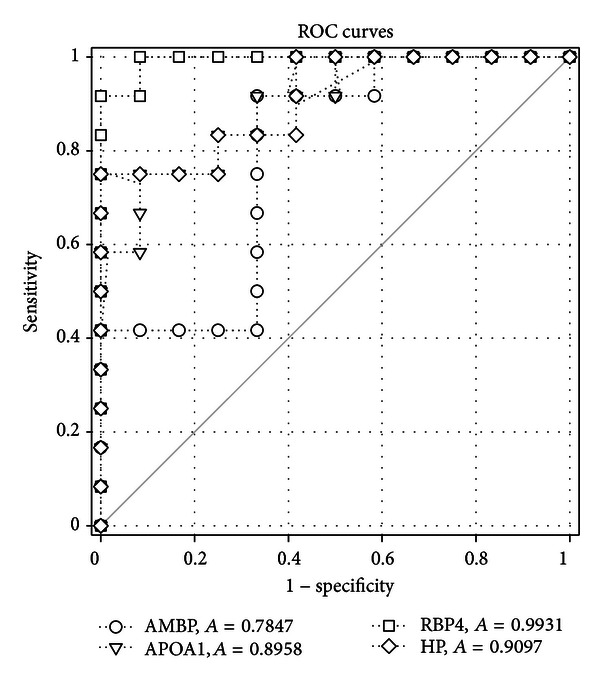
ROC curve analysis for AMBP, APOA1, RBP4, and HP in the peritoneal dialysate samples of DM patients. The estimated areas under the curves are 0.7847, 0.8958, 0.9931, and 0.9097, respectively.

Figure 2 shows the representation of the ELISA analysis results of peritoneal dialysate samples. The individual values and the means with standard errors for each protein were also showed in the Figure 2. In the peritoneal dialysate samples, AMBP, APOA1, RBP4, and HP were detected to be strongly downregulated in the DM patients (*P < 0.05, *n* = 12). The receiver operating characteristic (ROC) curve analyses showed that the differential proteomic features were potential protein biomarkers for DM (Figure 3). The areas of the ROC curve in the 95% confidence interval of AMBP, APOA1, RBP4, and HP were 0.7847, 0.8958, 0.9931, and 0.9097, respectively. 

**Figure 4:**
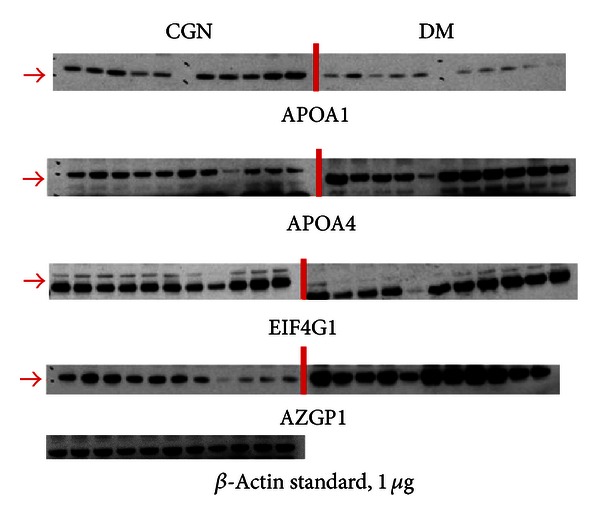
Confirmation of APOA1, APOA4, EIF4G1, and AZGP1 expressions by Western blot analysis. All peritoneal dialysate samples of CGN and DM patients were confirmed by Western blot analysis and the representatives are shown in this figure.

**Figure 5:**
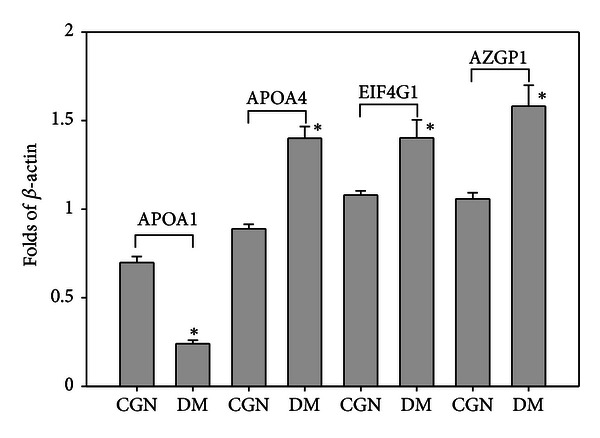
Protein expression in peritoneal dialysate samples of CGN and DM patients. The quantitative analysis of Western blotting was carried out using the ImageQuant-TL-7.0 software.

**Figure 6:**
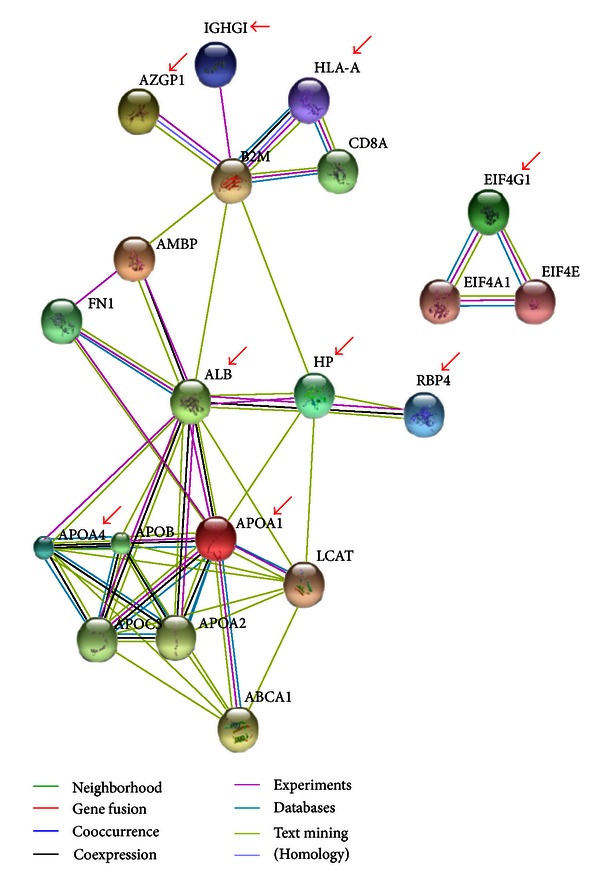
The protein-protein interaction pathways were performed. Proteins identified in this study were marked by red arrows.

The identified candidate proteins that may be related to DM are confirmed differential protein expression with Western blotting. Each peritoneal dialysate sample, containing 10 *μ*g of protein, was applied into a precast gel and transferred to a PVDF membrane.  Figure 4 shows the representation of the Western blotting analysis results of APOA1, APOA4, EIF4G1, and AZGP1 in the peritoneal dialysate samples. Compared with Western blotting of the *β*-actin standard, the concentrations of candidate proteins in peritoneal dialysate samples were detected and analyzed by using the quantitative analysis software ImageQuant-TL-7.0. The *P* values between CGN and DM were less than 0.05 (Figure 5, *P < 0.05, *n* = 12). The protein-protein interaction pathways were performed by String 9.0 Web software. Ten proteins identified in this study were marked by red arrows (Figure 6). 

Several identified proteins in this study were reported as peritoneal dialysate proteins, such as APOA1, HP, ALB, IGHG1, and AZGP1 [15, 19]. In our study, some proteins were also indicated that may relate to DM. 

HP is synthesized in the liver as a proprotein, processed by proteolytic cleavage into *α* and *β* subunits, and released into the bloodstream as a tetrameric protein. The expression of the HP subunits has caught the attention of investigators because of their exceptionally high degree of stimulation by cytokines and dexamethasone [20]. In this study, the expression of HP was downregulated in DM patients. HP is a defensive response protein, which may control the equilibrium between tolerance and immunity to nonself antigens. In addition, high blood glucose may cause chronic systemic inflammation. Thus, DM patients need to take medication to control blood glucose and inflammation, which may due to the downregulation of HP.

APOA4, a 46 kDa plasma apolipoprotein, is synthesized predominantly in the small intestine and is an antiatherogenic and antioxidative plasma glycoprotein involved in reverse cholesterol transport. The increased expression of APOA4 was found in chronic kidney disease (CKD) patients [21]. In previous studies, plasma APOA4 levels were increased in DM patients, especially in noninsulin-dependent diabetes mellitus (NIDDM) patients [22, 23]. The increase in expression of APOA4 may be related to hypertriglyceridemia (hTG), hyperglycemia, hormonal dysregulation, inflammation, and to a lesser extent to HDL cholesterol level [24]. However, the metabolism pathway and relationship between APOA4 and DM were unclear.

APOA1 is a component of the high-density lipoprotein responsible for the cholesterol transport into liver. APOA1 was associated with DM showing downregulation in DM patients [25]. Our 2DE, Western blotting, and ELISA results were consistent with those of previous studies [26–29]. 

RBP4 is a novel adipocytokine (adipocyte-secreted hormone), which shows upregulation in insulin-resistant animal models. In the clinical study, it is reported that the RBP4 level is higher in type 2 DM, gestational DM, and metabolic syndrome patients [30, 31]. However, in a study reported by Krill et al., it was found the RBP concentrations measured in DM serum samples were significantly lower than those in the healthy group [32–34]. Also, in another report, the mean serum vitamin A and RBP levels were significantly lower in the insulin-dependent diabetes mellitus (IDDM) patients. The linear regression between vitamin A and RBP levels was statistically significant [35]. In our study, the RBP4 level was downregulated in DM group. However, the role of RBP4 in DM is still challenged. 

There are not many reports about the role of AZGP1 in DM patients. AZGP1 is a candidate gene for age-dependent changes in the genetic control of obesity. In the study reported by Gohda et al., AZGP1 mRNA level was upregulated in the type 2 DM mice [36]. This is consistent with our experimental results.

AMBP is a glycoprotein also known as protein HC, present in body fluids. In the study reported by Shore et al., the level of AMBP in urine was significantly elevated in the NIDDM patients, which may be related to high blood sugar level and poor glycaemic control [37]. Although this is inconsistent with our experimental result, there are other reports which supported our data [38, 39]. 

The expression of EIF4G1 was upregulated in the DM patients in our study. Unfortunately, the literature mentioning the relationship between EIF4G1 and DM was unavailable. It may be worthy to reexplore this biomarker in a future study.

Previous studies have indicated that HP, AMBP, and AZGP1 were the candidate biomarkers for DM [40–42]. We also observed the changed levels of RBP4 and EIF4G1 in DM. In conclusion, those results support the hypothesis that those proteins may associate in the metabolism of DM. These proteins may not be sensitive diagnosis biomarkers for DM; however, they may be indicators of alteration in glycemic control.

## 4. Conclusions

Many investigations have focused on the development of biomarkers as a diagnostic tool. Proteomic approaches are powerful tools for analyzing proteins in complex mixtures. 2DE and MS analysis are useful for the analysis of human samples in a clinical research environment; they can be utilized when identifying the origins of samples of body fluids, analyzing protein phenotypes, monitoring disease processes, and searching for new disease markers.

There are only a few reports concerning proteomic analysis for the investigation of clinical peritoneal dialysate. This may be due to difficulties with obtaining “healthy” control specimens of peritoneal dialysate. Thus, in this study, we utilized the peritoneal dialysate from CGN patients as control samples and compared them with the DM peritoneal dialysate. Experimental results showed that ten proteins were differentially expressed in DM peritoneal dialysate samples. Although the sample number was not large, most of the proteins with differential expression in peritoneal dialysate samples were also found in plasma or serum. These proteins may not be new biomarkers; however, they may indicate a situation for possible drug treatment or glycemic control. In this paper, we may not be able to provide a final decision for the function or presence of these proteins, but these data support the need to continue and expand proteomic analysis of total proteins in DM peritoneal dialysate. The results of these studies still need to be verified by larger clinical studies. In conclusion, these proteins are valuable for the identification of differentially expressed proteins involved in the proteomics database and screening biomarkers for further study of DM.

## Figures and Tables

**Table 1:** The 10 peritoneal dialysate proteins identified with higher confidence levels (at least three unique peptide sequences matched) in this study.

Accession no.	Protein name	Expression	MW (Da)	*PI *Value	Mascot score	Match queries	Sequence coverage	Peptide
P02768	Albumin (ALB)	Downregulation	69367	8.2	66	4	17%	K.FQNALLVR.Y
								K.AVMDDFAAFVEK.C + oxidation (M)
								K.KVPQVSTPTLVEVSR.N

P02760	Alpha-1-microglobulin/bikunin preproprotein (AMBP)	Downregulation	38999	5.95	349	7	21%	R.ETLLQDFR.V
								K.WYNLAIGSTCPWLK.K + carbamidomethyl (C);
								R.GECVPGEQEPEPILIPR.V + carbamidomethyl (C)
								R.EYCGVPGDGDEELLRFSN.-
								R.VVAQGVGIPEDSIFTMADR.G
								R.VVAQGVGIPEDSIFTMADR.G + oxidation (M)
								R.ETLLQDFRVVAQGVGIPEDSIFTMADR.G + deamidated (NQ)

P02647	Apolipoprotein A-I (APOA1)	Downregulation	30778	5.39	244	12	44%	K.VQPYLDDFQK.K
								K.WQEEMELYR.Q + oxidation (M)
								R.THLAPYSDELR.Q
								K.VQPYLDDFQKK.W
								R.DYVSQFEGSALGK.Q
								R.VKDLATVYVDVLK.D
								K.VEPLRAELQEGAR.Q + deamidated (NQ)
								K.VEPLRAELQEGAR.Q
								K.LLDNWDSVTSTFSK.L
								R.QKVEPLRAELQEGAR.Q + Gln->pyro-Glu (N-term Q)
								R.QKVEPLRAELQEGAR.Q
								K.DSGRDYVSQFEGSALGK.Q

P01857	Immunoglobulin G1 Fc fragment (IGHG1)	Downregulation	36106	6.95	154	5	36%	K.NQVSLTCLVK.G + carbamidomethyl (C)
								K.FNWYVDGVEVHNAK.T
								R.EPQVYTLPPSRDELTK.N + deamidated (NQ)
								R.TPEVTCVVVDVSHEDPEVK.F + carbamidomethyl (C)
								R.WQQGNVFSCSVMHEALHNHYTQK.S + carbamidomethyl (C); deamidated (NQ)

P02753	Mutant retinol binding protein (RBP4)	Downregulation	23010	6.29	235	4	84%	-.FSGTWYAMAK.K
								-.FSGTWYAMAK.K + oxidation (M)
								K.KDPEGLFLQDNNVAEFSVDETGQMSATAK.G
								K.KDPEGLFLQDNNVAEFSVDETGQMSATAK.G + oxidation (M)

P00738	Haptoglobin alpha2 (HP)	Downregulation	45205	6.46	77	4	21%	K.LPECEADDGCPKPPEIAHGYVEHSVR.Y + 2 carbamidomethyl (C)
								K.AVGDKLPECEADDGCPKPPEIAHGYVEHSVR.Y + 2 carbamidomethyl (C)
								K.LPECEADDGQPPPKCIAHGYVEHSVR.Y + 2 carbamidomethyl (C); deamidated (NQ)
								K.AVGDKLPECEADDGQPPPKCIAHGYVEHSVR.Y + 2 carbamidomethyl (C); deamidated (NQ)

P06727	Apolipoprotein A-IV (APOA4)	Upregulation	45399	5.23	1109	19	43%	K.VNSFFSTFK.E
								R.LTPYADEFK.V
								R.LEPYADQLR.T
								K.ALVQQMEQLR.Q
								R.LTPYADEFKVK.I
								K.LVPFATELHER.L
								R.DKVNSFFSTFK.E
								K.LGEVNTYAGDLQK.K
								K.KLVPFATELHER.L
								K.VKIDQTVEELRR.S
								K.LNHQLEGLTFQMK.K
								K.LKEEIGKELEELR.A
								K.SELTQQLNALFQDK.L
								K.LGPHAGDVEGHLSFLEK.D
								K.SLAELGGHLDQQVEEFR.R
								R.ENADSLQASLRPHADELK.A
								R.QKLGPHAGDVEGHLSFLEK.D
								R.ENADSLQASLRPHADELKAK.I

P25311	Zn-alpha2-glycoprotein (AZGP1)	Upregulation	34259	5.71	739	24	48%	K.SQPMGLWR.Q
								K.CLAYDFYPGK.I + carbamidomethyl (C)
								K.WEAEPVYVQR.A
								K.AREDIFMETLK.D
								K.AYLEEECPATLR.K + carbamidomethyl (C)
								K.QKWEAEPVYVQR.A + Gln->pyro-Glu (N-term Q)
								K.QKWEAEPVYVQR.A
								K.QKWEAEPVYVQR.A + deamidated (NQ)
								K.AYLEEECPATLRK.Y + carbamidomethyl (C)
								K.YYYDGKDYIEFNK.E
								R.QDPPSVVVTSHQAPGEK.K + Gln->pyro-Glu (N-term Q)
								R.AKAYLEEECPATLRK.Y + carbamidomethyl (C)
								K.EIPAWVPFDPAAQITK.Q
								R.QDPPSVVVTSHQAPGEKK.K + Gln->pyro-Glu (N-term Q)
								R.QVEGMEDWKQDSQLQK.A + Gln->pyro-Glu (N-term Q)
								R.QVEGMEDWKQDSQLQK.A + deamidated (NQ); Gln->pyro-Glu (N-term Q); oxidation
								R.QVEGMEDWKQDSQLQK.A
								R.QVEGMEDWKQDSQLQK.A + deamidated (NQ)
								R.QVEGMEDWKQDSQLQK.A + oxidation (M)
								R.QVEGMEDWKQDSQLQK.A + deamidated (NQ); oxidation (M)
								K.NILDRQDPPSVVVTSHQAPGEK.K
								K.NILDRQDPPSVVVTSHQAPGEK.K + deamidated (NQ)
								K.HVEDVPAFQALGSLNDLQFFR.Y
								K.NILDRQDPPSVVVTSHQAPGEKK.K

Q04637	eukaryotic translation initiation factor 4A isoform 1 (EIF4G1)	Upregulation	175491	5.32	469	8	28%	R.QFYINVER.E
							K.GYDVIAQAQSGTGK.T
								K.GVAINMVTEEDKR.T
								K.MFVLDEADEMLSR.G
								R.DFTVSAMHGDMDQK.E
								K.LQMEAPHIIVGTPGR.V
								R.GIYAYGFEKPSAIQQR.A
								K.LNSNTQVVLLSATMPSDVLEVTK.K

P01892	Human class I histocompatibility antigen (HLA-A)	Upregulation	40922	6.46	129	3	36%	R.VNHVTLSQPKIVK.W
							K.SNFLNCYVSGFHPSDIEVDLLK.N + carbamidomethyl (C)
								K.SNFLNCYVSGFHPSDIEVDLLK.N + carbamidomethyl (C); deamidated (NQ)

**Table 2:** Subcellular location and protein function of 10 proteins with higher confidence levels identified in peritoneal dialysate of CGN and DM.

Accession no.	Protein name	Subcellular location	Biological process	Molecular function	Protein function
P02768	Albumin (ALB)	Secreted	Cellular response to starvation Hemolysis by symbiont of host erythrocytes Maintenance of mitochondrion location	Binding protein	Serum albumin, the main protein of plasma, has a good binding capacity for water, Ca2+, Na+, K+, fatty acids, hormones, bilirubin, and drugs. Its main function is the regulation of the colloidal osmotic pressure of blood. Major zinc transporter in plasma, typically binds about 80% of all plasma zinc.

P02760	Alpha-1-microglobulin/bikunin preproprotein (AMBP)	Secreted	Cell adhesion Heme catabolic process Negative regulation of immune response	Serine-type endopeptidase inhibitor activity Calcium oxalate binding Small molecule binding Calcium channel inhibitor activity	Inter-alpha-trypsin inhibitor inhibits trypsin, plasmin, and lysosomal granulocytic elastase. Inhibits calcium oxalate crystallization

P02647	Apolipoprotein A-I (APOA1)	Secreted	Cholesterol metabolism Lipid metabolism Lipid transport Steroid metabolism Transport	Binding protein	Participates in the reverse transport of cholesterol from tissues to the liver for excretion by promoting cholesterol efflux from tissues and by acting as a cofactor for the lecithin cholesterol acyltransferase (LCAT). As part of the SPAP complex, activates spermatozoa motility.

P01857	Immunoglobulin G1 Fc fragment (IGHG1)	Secreted, membrane	Complement activation, classical pathway Innate immune response	Antigen binding	G1m marker

P02753	Mutant retinol binding protein (RBP4)	Secreted	Glucose homeostasis Gluconeogenesis response to insulin stimulus	Retinol binding Retinol transporter activity	Delivers retinol from the liver stores to the peripheral tissues. In plasma, the RBP-retinol complex interacts with transthyretin; this prevents its loss by filtration through the kidney glomeruli.

P00738	Haptoglobin alpha2 (HP)	Secreted	Defense response Metabolic process	Catalytic activity	Haptoglobin combines with free plasma hemoglobin, preventing loss of iron through the kidneys and protecting the kidneys from damage by hemoglobin, while making the hemoglobin accessible to degradative enzymes. Uncleaved haptoglobin, also known as zonulin, plays a role in intestinal permeability, allowing intercellular tight junction disassembly, and controlling the equilibrium between tolerance and immunity to nonself antigens.

P06727	Apolipoprotein A-IV (APOA4)	Secreted	Cholesterol efflux Cholesterol homeostasis Cholesterol metabolic process Chylomicron assembly Chylomicron remodeling High-density lipoprotein particle remodeling Hydrogen peroxide catabolic process Innate immune response in mucosa Leukocyte adhesion Lipoprotein metabolic process Multicellular organismal lipid catabolic process	Antioxidant activity Cholesterol transporter activity Copper ion binding Phosphatidylcholine binding Phosphatidylcholine-sterol O-acyltransferase activator activity Protein homodimerization activity	May have a role in chylomicrons and VLDL secretion and catabolism. Required for efficient activation of lipoprotein lipase by ApoC-II; potent activator of LCAT. Apoa-IV is a major component of HDL and chylomicrons.

P25311	Zn-alpha2-glycoprotein (AZGP1)	Secreted	Antigen processing and presentation Cell adhesion Immune response Lipid catabolic process Negative regulation of cell proliferation	Fatty acid binding Protein transmembrane transporter activity Ribonuclease activity	Stimulates lipid degradation in adipocytes and causes the extensive fat losses associated with some advanced cancers. May bind polyunsaturated fatty acids.

Q04637	Eukaryotic translation initiation factor 4A isoform 1 (EIF4G1)	Cytosol	Cell death Insulin receptor signaling pathway	DNA binding Translation initiation factor activity	Component of the protein complex eIF4F, which is involved in the recognition of the mRNA cap, ATP-dependent unwinding of 5′-terminal secondary structure and recruitment of mRNA to the ribosome.

P01892	Human class I histocompatibility antigen (HLA-A)	Golgi membrane Plasma membrane	Type I interferon-mediated signaling pathway Regulation of immune response Interspecies interaction between organisms	Binding protein	Involved in the presentation of foreign antigens to the immune system.
